# On the Uses and Properties of Some Remedies

**Published:** 1830-07

**Authors:** John Gardner

**Affiliations:** Surgeon, &c.


### ON LEAD.

### *On the Uses and Properties of some Remedies.*

### By John Gardner, Surgeon, &c.

*Preliminary Observations. Modus Operandi of Morbid and Remedial Agents.*

In the preliminary remarks to these essays, I have endeavoured to show the necessity that exists for some improvement in the spirit of our investigations, especially for the exercise of minute observation and a more accurate discrimination of the phenomena observed, before we proceed to generalize our knowledge. In the employment of remedies, we are to look for the symptoms indicative of the specific agency of each substance, and, noting accurately their primary and secondary, their essential and accidental effects, endeavour to establish their exact uses and value.

In addition to what I then advanced in favor of a more philosophical method of research than has yet been adopted in therapeutics, I would suggest that the several sciences which constitute medicine have not thrown that light upon each other, which, from the nature of their relation, we should be justified in expecting: for nearly all our most esteemed remedies were introduced into practice by some circumstances independent of scientific research; and many of those which were employed in the infancy of physic have held their ground through all the progress of anatomical and physiological knowledge, and amid every revolution of pathological doctrine. Each school of pathology, however, which has successively flourished, has thought it necessary to explain, in accordance with its own peculiar tenets, the *modus operandi* of remedies, and to invent terms for expressing their supposed modes of action. From this cause, all our treatises on Pharmacy and Therapeutics abound in technicalities and contradictions; and there is no doubt that to the same cause we may justly attribute their palpable errors and deficiency in useful information. But, as a first step in the acquisiton of such knowledge as will be applicable to this as to every practical art, it is essential that we should entertain correct views of the precise objects of inquiry, and that our researches should be conducted according to the strictest rules; then we may be able to apply correctly the discoveries in the collateral sciences for mutual elucidation and illustration. The general assertion, that experience, and experience alone, must be our guide in the employment of remedies, is universally acknowledged to be just; but it by no means follows that what is implied in the word experience, as applied to this science, is understood. It is too often considered synonymous with experiment. Indeed, we think it still necessary to urge that, before any benefit will arise from *experiments* in the art of healing, a foundation must be laid by the acquisition of correct physiological knowledge, an improved system of pathology, and a more minute and comprehensive symptomatology. Then, and not till then, can a superstructure of experimental facts be reared, and therapeutics assume the stability and exactness of other physical sciences. Our discoveries in physiology and pathology are really only useful to us in so far as they are employed to enlighten our path, to direct our inquiries concerning the properties of remedies. The following views of pathology will, it is presumed, accord with these remarks, and illustrate what is to succeed on the properties of lead.

It is only of recent discovery that the seat of the vital principle in the animal economy, the nervous system, is made up of parts having distinct and peculiar functions. These parts, while they are woven into one whole, and appear to derive their powers from their combination, manifest their peculiarities at the point of distribution. Thus we find a separate nerve appropriated to each sensation, to voluntary motion, to the respiratory motion, to every other motion having an especial design (as in some of the muscles of the eye), to digestion, and it is more than probable to each peculiar secretion. Every healthy action has its origin in the nervous system, and it would appear in a part only of that system, a nerve. Now, there can remain no doubt that, whatever impression is made on the system by external agents, or whatever morbid action may arise, the primary seat of that impression, of that action, is some part of the nervous system: and this is equally true whether the agent operating be introduced through the channel of nourishment, immediately into the circulation by a vein, through the skin, or by the lungs in vapour; and it is seldom that the influence of any agent is exerted over the whole system; it is generally confined to some one nerve, evidenced by the disturbance of one separate function; in other words, it is local. This local effect corresponds with our present knowledge of the peculiar endowments of separate nerves. In many instances it is evident enough from its being manifested in a nerve having a function quite peculiar to itself, at other times there is a semblance of general disease, from several nerves with the same function in different parts being affected simultaneously. Although it is not essential to the truth of these remarks that affections of the whole system should not occur immediately from various causes; yet these general diseases are certainly explicable by the known laws of sympathy; the morbid action running in a consecutive series of associated organs, until it is no longer to be ascertained where was the primary disturbance. But, laying aside the consideration of causes for a moment, let us look at diseases as they appear in themselves. In every case of complication in the phenomena we are endeavouring to investigate, it assists us to contemplate the simplest possible in the first instance, and from them to advance to the more complex. Thus, therefore, in pathology, if we look for the simplest cases of disease, they will be found to be,

First, a morbid sensation; the seat of which, no one can dispute, is a nerve. This morbid sensation may vary in degree from one that is scarcely felt, and through every grade increase to a pain amounting to agony, still remaining a simple morbid state, a mere nervous sensation. Such a sensation accompanies, or rather precedes, almost every case of disease: in some instances the vascular changes so rapidly succeed, that we are unable to detect the sequence; in others, it would appear the vessels of the part do not sympathize, as the morbid sensation is not followed by any other changes.

Secondly, there may be a simple deprivation of power in a nerve of sensation, or in a nerve of motion, in short, in any nerve whatever; and a loss of feeling, a loss of motion, or a cessation of function, is its immediate evidence.

Thirdly, an increased power or sensibility to impressions, is also a simple state of disease, and nerves of all kinds are susceptible of it, manifested by increased intensity of feeling, spasm, and other violent actions.

Fourthly, to these we may doubtless add (as consisting of a morbid affection of the nerve,) those cases of depraved secretion, of which nothing more is at present ascertained than the simple fact of the formation of an unusual substance.

Each and all of these several states occur to individual nerves, or to many at the same time in the system, and, by coexisting, form complicated diseases, of sufficient energy to destroy life, and are yet obviously explicable on the supposition of an entire absence of vascular or organic derangement. No such changes can be displayed by the aid of the dissecting knife, and the hypothesis which assumes their existence is only supported by false reasoning on analogy. In the series of actions that constitute other complicated diseases, the first link in the chain is that which deserves the most minute attention in the study of causes: for, as all agents exert a specific influence, and generally on some local organ through the nervous system, this primary phenomenon will characterize the morbific agent. In inflammation, for instance, the train of symptoms marking its origin points unequivocally to the nervous structure of whatever organ it may attack, as the seat of the first impression, the beginning of the disease; and the causes operating in the production of this very frequent state of disease, the nervous symptoms vary, and the whole succession of changes vary also. The study of pathology must be based on a strict investigation of the numerous causes of disease, and their specific and essential effects; then of the laws of the transmission of morbid action from organ to organ, including what is usually termed sympathy, but in a more extended sense: and then of the effects left in the various structures of the body, which constitutes morbid anatomy. It is very questionable whether all the interstitial changes which leave evident derangements of organization, are the result of an identical action, or whether there are not various vascular actions, having no analogy with each other. The prevailing doctrine is, that they are the result of an identical action, and the term inflammation is applied to it. But, from the knowledge physiology supplies of the variously endowed parts of the nervous system, a variety of actions appears most likely; and this probability is increased by a reference to symptoms: for, even among those cases universally considered to be inflammation there are extremes, possessing no single symptom in common.

Taking into consideration the nervous diseases above described, the known specific effect of *some* morbific agent, and the doubtful nature of the evidence supplied by morbid anatomy, it would appear that pathologists are little likely to advance the healing art by attempting to generalize into one single state all diseases whatever: yet this is what our pathologists universally do, some only in effect, whilst others, and those of the greatest repute, distinctly aver that their aim is only the generalization of a fact, namely, that all disease consists essentially in inflammation. They thus utterly set at nought the true principles of the philosophy they profess to follow, which rests all its claims on logical induction from facts furnished by experience. It is altogether impossible to adopt one single principle to explain all the varied elements of knowledge contained in the functions of living bodies and their diseases. Newton, whom these pathologists desire to imitate, discovered the applicability of a general fact, but it was only to the simple *elements*, motion, and its modes, direction and velocity, that the law of gravity is adapted. No single fact can explain the affinities even of inanimate bodies. How much less likely is any one principle to explain all the phenomena of disease! Look at the diversity of the causes capable of disturbing the multifarious functions of the animal economy, their varied nature, material and mental; and if each cause is capable of an invariable effect, how few of these effects have as yet been ascertained? The research for a general fact in pathology will be equally futile with that for the philosopher's stone; and that such should still be followed, is a striking and sufficient proof that in this branch of medicine, (as I have before attempted to show with respect to therapeutics,) a more philosophical spirit, a more correct and systematic logic, is essential to its successful pursuit, and especially to such results as illustrate the *modus operandi* of remedies, and thus advance and systematize the healing art.

2. *Lead.*

There cannot, perhaps, be found a better example of the inefficient and unsatisfactory manner in which the study of remedies has been pursued, than is afforded by the history of lead. This metal, in some form or other, has been employed in the cure of diseases from the earliest ages to the present day, and yet it would be impossible to select one fact concerning its agency on the body in disease, which has been indisputably established. Indeed, an extraordinary prejudice has long prevailed against it, and even at the present day is entertained by a large proportion of medical men. The origin of this prejudice it is by no means difficult to trace; that it should still exist, is not so easily explained. The extensive employment of lead in a great variety of forms in the arts and manufactures necessarily brings often into notice cases of its baneful effects. Metallic lead is employed more than any other metal for uses that bring it into contact with almost every article of food and drink, in situations highly favorable to its oxydisement, and often for vessels to contain acid fluids. Small portions are dissolved, and, from the known effects of large quantities of menstrua in increasing the power of agents, we may learn why a deleterious effect is produced by cyder, light wines, &c. in which lead is dissolved. The oxides of lead, lytharge (protoxide) and minium (peroxide), the chloride (patent yellow), and the carbonate (white lead), are all employed by artisans, especially painters, in very large quantities, and in such a manner as to expose large surfaces of the body to their constant contact. Added to which, from a want of cleanliness, much is often taken into the stomach with the food; but, above all, in combination with turpentine, varnishes, and other volatile substances, it finds its way into the system through the lungs, and in a state of great activity. In all these cases we need not wonder at the occasional occurrence of colic. It may be questioned whether there is any other metallic substance, except iron, that could be so much employed with equal impunity: yet it is on this ground, and on this alone, that a prejudice has so long existed against the employment of every form of lead as a remedy. But experience teaches us, in the analogous instances of iron, mercury, antimony, arsenic, copper, silver, &c., that, while a metal in a state of purity may be, as to any power over the human body, altogether inert and useless, its combination with other substances, oxygen, chlorine, iodine, or in the state of salts, furnishes us at the same time with our most valuable remedies, and the most dangerous and powerful poisons. The attempt to bring into discredit the protochloride of mercury, on the ground that the perchloride, or the sulphate, of that metal is poisonous, would be looked upon as the height of absurdity. But, in the case of lead, this new species of logic is admitted as conclusive: many writers on therapeutics have considered it quite sufficient to show that some salts have certainly produced colic, to induce them to deprecate the use of every form of the metal as a remedy.

The authority of Sir George Baker is generally referred to, as conclusive evidence for the dismissal of lead from our materia medica. His papers, published in the first volume of the Medical Transactions, are intended to prove that colica pictonum is in every instance to be traced to the introduction of lead, in some form or other, into the system; and much ingenuity and erudition are displayed in conducting the argument (for an argument it is), laboured to establish a preconceived hypothesis: and as, alas! too often happens in disputation, the authorities he refers to, if examined, will be found not to warrant his statements; indeed, they sometimes stand in direct opposition to them, and he unfortunately suppresses all the evidences against his views. Having once determined to prove that colic and lead are inseparably connected, he, of course, recommends its disuse as a remedy, and disbelieves all the numerous authors who speak of it favorably.

The etiology of that interesting disease, colica pictonum, has not yet been satisfactorily explained. Its prevalence among painters has given rise to the idea that it is connected with lead, In Derbyshire, where it is frequent, leaden vessels are used to contain cyder, and it is certain that a malate of lead, in large proportions of menstruum, does occasionally produce colic. But, if we reflect on the comparative infrequency of the disease in connexion with the extent to which the metal is employed, we shall certainly see that the constancy of a connexion between them is problematical. There are other facts pointing to different causes for the disease. It is endemical in some of the West-India islands, where no lead is employed; and we know that it is produced by the oxides of other metals. From the frequency with which lead is impregnated with arsenic, perhaps that metal may be looked upon as the most common cause. At the same time, we do not mean to deny that the carbonate and some other forms of lead will produce colic, and there can be no doubt that many constitutions are so susceptible of their influence, that even the milder salts cannot be given with impunity. In no other instance has this circumstance driven a remedy from practice. Were such a principle adopted, we might lay aside every active drug in the Pharmacopœias.

Another writer on the subject is referred to in the Dispensatories, to deepen the existing prejudice against lead. In the "Journal de Physiologie experimentale" of Magendie, the third Number, July 1821, is a paper on Lead, by Dr. Gaspard; a quotation from which will show the credit that is due to the references made by compilers of Dispensatories to confirm their own dogmatic assertions. "Un medicin," says the author of that paper, "que j'estime beaucoup, et dont je loue le zèle à augmenter les richesses de la matière medicale, et a determiner les proprietés réelles des medicamens, m'a etonnée en publiant dernierement, comme resultats de ses recherches experimentales sur l'acetate de plomb: 1. Que ce sel peut être administré sans danger aux phthisiques, à la dose continuée de douze grains par jour; 2. Qu'on lui a reproché a tort de produire la colique dite de plomb." M. Gaspard proceeds to relate his experiments, which he conceives invalidates these conclusions. He injected into the veins of three dogs a solution of acetate of lead, which, being several times repeated, at length destroyed the animals. On throwing it into the cellular tissue of a cat, he produced an abscess difficult to heal. He next placed some tadpoles into a solution of the acetate of lead; they lived for some time, but at length, after many contortions, minutely described by the writer, they died. "Je conclus," he continues, "de ces experiences, 1, Que l'acetate de plomb introduit, meme à trés petite dose, dans les voies circulatoires, est un poison tres dangereux, quoique lent et insidieux; 2, Qu'il ne peut être employé à l'Interieur, sans danger, par un medicin prudent, à quelque dose que ce soit." Such is the conclusion, almost literally translated by an English writer, as an unquestionable truth, who refers to M. Gaspard as having justly established it.

Physiologists have always been too much disposed to apply principles deduced from observation on the lower animals, to explain the phenomena of the human body, in the face of the well-known fact that substances from which animals derive a wholesome nourishment are to our species deadly poisons; and, on the contrary, some of our most useful remedies are fatal to brutes. The details M. Gaspard gives us of the morbid appearances on the dogs, are utterly worthless; for, even supposing the effects of lead upon the human body analogous, they would doubtless be very different, introduced directly into the circulation by the veins, to those resulting when the substance is taken into the stomach, and there elaborated in the process of digestion.

Cases of death from poisoning by any form of lead, are either very rare or they have not been recorded; at least, English medical literature furnishes very few. In such cases as have been properly investigated, no morbid changes of structure whatever could be traced.*

We have alluded to the misstatements of the most esteemed works on Materia Medica, concerning the properties of the salt of lead: it may be proper to quote the most influential to justify this charge.

Cullen says, "Hardly any practitioner will think of employing lead as an internal remedy." Indeed, he entertained so decided a prejudice against lead in every form, as equally to condemn its employment externally: he praises a very worthless book of a Mr. Aiken, written against the well-known tract of M. Goulard. Who at present can doubt of the value of the subacetate of lead as an external application in inflammation?

Duncan, in the Edinburgh Dispensatory, condemns its use altogether, and asserts, without alleging any reason, that "it is entirely to be rejected."

A. T. Thomson, in the London Dispensatory, remarks that, "taken internally, acetate of lead is a very powerful *astringent* and *sedative;* combining it with opium prevents the deleterious effects which salts of lead are apt to produce when taken into the stomach. The dose should not exceed half a grain every six or eight hours."

Murray observes, that, "From the deleterious agency

* Medical Society's Transactions, vol. i, part i. The morbid appearances produced by lead are described by Dr. Christison, p. 422. (*Christison on Poisons.*)—Ed.

of lead on the system, it is a remedy which must be used with reluctance, and which is accordingly scarcely ever used in modern practice.

With similar cautions it is spoken of by Brande, Paris, Ure, Cox, Chapman, and a host of writers on the practice of physic; all, however, agreeing in one error, namely, the assumption that, because some salts of lead are deleterious under some circumstances, *therefore* all must be equally so. It is true also that all the encomiums that have been lavished on the medical virtues of lead, except by very recent writers, were applied equally to all its salts; and when, in collecting evidences of their injurious effects, they were all equally confounded, we cannot be surprised that a prejudice should be, in deference to those authorities, widely spread. And as the forms of the remedy, so also the diseases in which it was employed were never distinguished by any judicious test. All the forms of lead were employed so late as the middle of the last century, particularly in Holland, Denmark, and other countries of the continent. Authors recommend them in so great a diversity of cases as to afford De Haen ample room to make out an appearance of reason against their use. To him we may certainly refer the idea of Sir G. Baker, of finding in lead an invariable cause of colic, and of depreciating its employment as a remedy. In vain Dr. Seerap defended it in a separate treatise: the superior reputation of those who opposed it prevailed, and the result, as we have seen, continues to extend its influence to the present day.

Within these few years, however, numberless writers have laboured to prove that the acetate of lead may be employed with caution, and in combination with opium, in very small doses; but no one has paid sufficient attention to the subject to establish its true value, and enable practitioners to prescribe it in effective doses with confidence. There are certainly many facts concerning its agency on disease not at present known. If these hints should lead to more successful investigations, the end of the writer will be answered; yet something, he thinks, both new and useful will be found in what follows.

In the first place, the acetate of lead, taken into the stomach even in considerable quantity, produces *no immediate effect whatever*,* that is, in the quantity of one drachm to

* Dr. Christison (p. 413 *loc. cit.*) states that he has often given the acetate of lead in divided doses, to the amount of eighteen grains, for eight or ten days, without remarking any unpleasant symptom whatever, except once or half an ounce; in this respect agreeing with many of our most esteemed drugs, ergot, sarsaparilla, &c. It is remarkable that this fact should have so long escaped notice. We constantly read of great alarm being excited by the acetate of lead taken accidentally, and of violent means had recourse to for removing it from the stomach. Many cases are recorded in the early volumes of this Journal, in the Medical Transactions, and other periodicals, of its having been taken in large quantities without producing any deleterious effect. In one instance recorded in the former Journal, a whole family partook largely of some broth in which nearly a pound was dissolved; and the escapes in all such instances are mentioned as very marvellous. A paper appeared lately in the Lancet, detailing experiments performed on the author's own person, with a similar result; and as, at a medical society, it was stated that the acetate of lead produced no effect unless some decomposition took place in the stomach, we expect to hear it condemned as a remedy, not on account of its poisonous properties, but because it is altogether inert. There are, however, persons who are affected by acetate of lead in a most peculiar manner; that is, it produces a decided tendency to colic in a very short space of time: but such cases are very rare, and we must be content to say of them that they depend on the idiosyncrasy of the individual. On this account a caution is necessary in using it, which we shall presently explain. Every one must be familiar with cases where the most innocuous substances cannot be taken, or even smelt, with impunity. This will consequently have no weight in determining the propriety of the use of acetate of lead as a remedy.

Secondly. The best known of the secondary effects of the acetate of lead, is its tendency to check inordinate discharges, either of blood or secreted fluids. It is as an astringent merely that it has been so generally employed; some practitioners extolling it very highly, while others are unable to observe any advantage from it. The continental writers greatly extolled all the salts of lead in hemorrhagies from the lungs, stomach, and uterus; and immediately after the papers of Sir G. Baker in the Medical Transactions, we find papers by Dr. REYNOLDS and Dr. LATHAM, twice slight colic. VAN SWIETEN* even mentions a case in which it was given to the amount of a drachm daily for ten days, before it caused any material symptom.—ED.

* Comment. 1060, t. iii. p. 347. Editio Dan Barbari.

highly extolling its effects "in hemorrhagies, in colliquative diarrhœas, and hectic perspirations, and especially in the semi-purulent expectoration which too often terminates in pulmonary ulceration and consumption." Dr. Latham asserts his belief "that there is some fallacy in imputing to lead the deleterious qualities almost universally attributed to it." It has also been recommended, by good authorities, in ordinary diarrhœa, in dysentery, in leucorrhœa and in gonorrhœa, in diabetes, &c. It would be too much to expect that in all these diseases, occurring as they do under so great a diversity of circumstances, any one remedy should be constantly beneficial. There are certainly cases in which the acetate of lead is an invaluable remedy, and others in which it is worse than useless. The pathology of hemorrhagies has not yet been sufficiently investigated to establish accurate distinctions between these; but it is certain that, in plethoric habits, where there is great muscular strength and strong vascular action, the administration of lead should never precede venesection: perhaps in these cases there is no need of it, as general and local bleeding and strict antiphlogistic regimen are sufficient. In cases occurring in weakened constitutions, especially where there is also great morbid sensibility and irritability, it is beneficial. In dysentery, the irritation of a part of the lower intestines, induced by the hardened feces, cannot always be allayed by opium, and it is desirable to do this before we have recourse to purgation. Now this irritation, together with the consequent flux of blood, is more speedily relieved by the acetate of lead than any other remedy: it should be given in larger doses than are usually employed, and followed up, in the ordinary manner, by purgative medicines. That many other discharges, particularly gonorrhœa, leucorrhœa, &c., are relieved by the acetate of lead, will be readily believed from what we know of its effects on the minute vessels; but the length of time necessary to ensure its effects, and the more ready means of cure we possess, will perhaps preclude its becoming a common remedy.

Thirdly. A direct influence upon the generative organs is apparently justly attributed to lead. GALEN* mentions this property, "ad coercendum et delendum libidinum." PARACELSUS extols it as a remedy in some forms of madness;† and a remarkable case of nymphomania is related in the Medical Repository, vol. iv. p. 256, cured by the admi-

* Tenth book of Simple Medicines.

† De Morbis Amentium.

nistration of the superacetate. The property of this salt of lead here alluded to was well known on the continent; for De Haen observes, "Sacchari saturni usus internus laudantur, quia refrigerat et exsiccat, semen extinguit et venerem flaccescentem inducit."* The only allusion to this property I know of in English medical literature, is the case above referred to. It certainly offers an important prospect of usefulness in cases of madness induced by ungovernable animal passion. Such cases are by no means rare, and the failure of general means of cure would render any chance of a resource desirable.

Fourthly. The most important property of the superacetate of lead, however, is its influence on nervous pain. I am not aware that this has been observed before: it suggested itself from reflecting on the symptoms produced by the salt when exhibited for a long period in hemorrhagies; and I can confidently announce it, after a long experience of its effects, to be the most valuable remedy we possess for pain of a nervous character, unconnected with vascular or organic changes; in a word, idiopathic pains. One of the best known instances of this affection is the tic douloureux. This formidable disease, there is no doubt, exists in two kinds: first, as a simple nervous affection; and, secondly, as symptomatic of organic lesions of the brain. The best pathologists have been unable to distinguish the one from the other, except the death of the patient gave the opportunity for a rigorous examination. The disease is comparatively rarely fatal: it more frequently exists for months, or even years, a source of disquietude and torment to the unhappy sufferer. That this nervous pain is not confined to the nerves of the face, is universally known: it occurs in every part of the body, in the superficial ramifications of the nerves, and the general term neuralgia is applied to it. Attempts have been made by some writers to show that it always consists in inflammation of the tunics of the nerves, and by others to resolve into this disease many other and dissimilar painful affections: but, while we think this generalization erroneous, we have been led to entertain the opinion that a similar morbid state of the nerves, perfectly analogous to that existing in superficial nerves, and termed neuralgia, frequently occurs in the internal parts of the body; in the nerves of the chest, liver, gallbladder, stomach, spleen, and other parts. There are few chronic affections so prevalent as local pain in the chest and abdomen, often in the obvious

* Ratio Medendi pars decima.

situation of an organ, but most frequently so situated that we are unable to determine its precise seat. Such pains, unattended by fever, continue for months, or even years, aggravated at uncertain intervals, and resisting the influence of all our known remedies, until perhaps some decisive change in the condition of the patient, in his manner of living, from other and more active disease, from mental emotions, change of air, or some other circumstances, the pain is lost. In these cases we may in vain ring every possible change on the depletory measures, purges, mercurials, &c., ordering the patient from town to country, and from country to town, and, after all, have the mortification of seeing him suffer as much as at the beginning. To every one in extensive practice, such instances are often presented; and a safe and efficacious remedy must be a great desideratum. The acetate of lead is such a remedy, and it may be given to as great an extent, under proper precautions, as any metallic substance we employ. It appears to act by allaying the morbid sensibility of the extremities of the nerves immediately, and not through the medium of any change in the circulating system. This power is not analogous to that possessed by any other of the class of remedies we term sedatives, but is a peculiar and specific effect of the acetate of lead. A few cases, selected from a great number, will illustrate this, and point out the precautions necessary to be observed in its exhibition.

### Case I. *Nervous Pain in the Chest.*

E. J., a robust young woman, of a florid complexion, twenty-one years of age, has been subject to a pain in the chest for about nine months: it has been constantly felt in a slight degree, but severe paroxysms have occurred several times. The situation of the pain is described as being most severe in the middle of the breast bone, but spreading over a large space of the chest. No obvious cause can be assigned for the recurrence of a paroxysm. During the intervals, and even at first, when the pain is most severe, the pulse is not at all affected, being seventy-five, without hardness, fulness, or contraction; the tongue is clean, the skin moist; and, indeed, every function of the body is well performed. The pain continues severe; but, a short time before, the breathing becomes hurried, the pulse quick and irritable, the tongue furred in the middle, with redness of the edges, the countenance flushed; and there is a frequent, distressing, and dry cough. These symptoms are severally aggravated, the pain increases, and in about twenty-four hours the aspect of the disease would apparently warrant its being termed inflammation of the chest. At the period of the first attack, the paroxysm had existed for some hours when I was called in to see the patient, and I regarded it as an inflammatory affection, and treated it by large bleedings. The blood first drawn did not exhibit any buff, but at the third bleeding there was a slight appearance of it. The pain was little affected. Leeches were applied, blisters and the strictest antiphlogistic regimen enforced; but the influence of these measures on the disease was by no means satisfactory. After about ten days the pain subsided into a dull aching sensation; and this I could not relieve by any plan of diet, or by medicines, sedative, tonic, or purgative. Mercury also was tried in vain.

After about two months, another severe paroxysm, with a precise reiteration of the symptoms, was treated on the same plan, and with a result perfectly similar. The pain remaining after the severer paroxysm had subsided was sufficient to give the patient great annoyance, and to cause a constant depression in her spirits.

A third attack occurring, I was sent for sufficiently early to see the successive changes in the symptoms, and remarked that all had their origin from the pain. This time stimulants and narcotics were depended on, with such variation as the circumstances suggested; but this plan succeeded no better than the former. The dull harassing pain left was accompanied by no deviation from a healthy state of the functions, except rather a feeble appetite and sluggish bowels; the latter readily obviated by any mild aperient. Change of air, perfect rest, then exercise and various modes of living, were tried, and without any apparent effect on the pain.

In another month the attack returned, with more than ordinary severity: having then recently seen the effect of acetate of lead in cases of nervous pain, I gave three grains of it every three hours, followed by a draught of very dilute acetic acid, (ten minims of the strong acetic acid to two ounces of water.) In about thirty-six hours the pain was greatly relieved; more strikingly so than it had ever been by former remedies. Then, extending the interval of the doses first to four, and afterwards to six hours, it was continued for four days longer, when the pain was completely removed, and not the slightest vestige of it has existed since, and the patient perfectly recovered her health and spirits.

In administering the acetate of lead, it is necessary to be aware that, in some constitutions, the first few doses may produce a griping pain in the bowels: this has, however, happened but in one or two instances in my practice, and was immediately relieved by a small dose of sulphate of magnesia. Costiveness is a more frequent effect, and happens after the acetate has been taken for a day or two: it always may be readily removed by some mild purgative, castor oil, jalap, rhubarb, &c.; but all salts should be avoided. While the acetate of lead is taken, the stools are black, making it apparent that some of the lead must get into the larger intestines, and be combined there with sulphuretted hydrogen. After continuing it for a period varying in different individuals, from a few days to a week or more, it appears to act on the nervous system specifically, the disease yields, and a numbness is felt in the ankles and wrists, attended with shooting pain; the evidence that the influence of the medicine is exerted on the system. These effects, resulting from a slow exhibition of the acetate of lead, as compared with the comparative inertness of a single dose, closely resemble the variable uses of other remedies, (calomel or colchicum, for instance,) according to the mode of their employment. When these symptoms occur, the medicine should be discontinued; but, indeed, it will seldom be found necessary to proceed further.

Case II. Mrs. H., a pale but stout female, aged thirty-five, had lost all her children from scarlet fever, and, with her husband, had herself gone through a severe attack of the disease, and suffered much from the sequelæ, œdematous swelling of the legs and other parts. Before her strength could be restored, she was attacked by a pain in the face, at first slight in degree and constant, but it soon became aggravated and intermittent: in a word, it was unequivocally tic douloureux. It is unnecessary to describe the paroxysms of this well-known affection, and the causes exciting them in greater or less severity: suffice it to say, the patient's sufferings were *tremendous.* The carbonate of iron and sulphate of quinine were severally tried, and fairly, but without the least apparent benefit. I then had recourse to the acetate of lead, exhibited in the same manner as in the preceding case. The numbness and pain in the joints were produced in five days, and the pain in the face was entirely removed. Aperients were called for during the administration of the remedy, and for a short time afterwards, which, with the assistance of a light tonic, restored the patient to her accustomed health.

Case III. The next case I shall quote tends rather to illustrate the views I have ventured to give in pathology, than to be decisive of the value of the acetate of lead. It was, indeed, the first case in which I employed that remedy for pain of a nervous character, directed by reflections on the effects usually seen when it has been given in hemorrhagies, and one or two cases of gastrodynia relieved by it.

A young man, about eighteen years of age, applied to me for relief from a pain in the right side. The history he gave of his disease was, that it had occurred first when he was at school, about four years before; that he could scarcely ever say he was entirely free from it, but that, at uncertain intervals, from a day or two to several weeks, he was liable to pain of extreme severity. He had been treated by several medical men, and, as far as I could learn from his own relation, on the general principles of bleeding, purging, counter-irritants, &c., but he had never experienced any decided benefit from any thing. The pain was situated exactly in the region of the gallbladder, chiefly in a space of about three fingers' breadth, but spreading around into a larger space. There was no tenderness on pressure, no evidence of any disturbance in the function of the gallbladder, liver, stomach, lungs, or bowels; the pulse quickened during a paroxysm, the tongue became furred, and skin hot and dry; but at other times no marks of febrile action were present. The patient's appetite was good, and there was no sickness; he followed a laborious occupation, that of a butcher, ate heartily of animal food, and was capable of much muscular exertion.

His first application to me was in November 1827. He had been accustomed to seek the temporary relief of an opiate, and strong pressure on the part, after a blister and a free purgation. I gave him the acetate of lead in small doses, and combined with opium: this not relieving, I ventured on larger doses and uncombined, and it succeeded beyond my expectation. The patient felt more benefited by this than any former plan, and in a fortnight he was free from pain.

In March 1828, he had again a slight attack, but it was immediately subdued by a few doses of the remedy. From this time he continued well until the 17th of December in the same year, when, after feeling a slight degree of pain for a few days, an attack of extreme severity suddenly came on.

No small portion of the public unfortunately entertain the extravagant notion that a remedy, to be of any value, must not only cure a present malady, but must prevent the possibility of a future return, in spite of every opposing habit and circumstance of the patient. In the present instance, as I was not immediately at hand when summoned, a physician was sent for: I communicated my opinion of the nervous character of the disease to him, but he saw reason to adopt a different view, and treated the case as inflammatory, by bleeding, purgatives, salines, cupping, leeches, blisters, warm baths, and every other means that could be devised, and without the slightest benefit. Indeed, as the patient became weakened, the paroxysms of pain became more frequent and severe, and at length there was scarcely any intermission. Sedatives, opium, and henbane internally, belladonna in the form of extract externally, were equally unsuccessful. Again, the depletory measures were gone over, and a period of three months had now elapsed since the first commencement of the attack; and, although the strength of the patient was by this time dreadfully prostrated, the patient being put under my care, I determined on a trial of the acetate of lead. After three days the pain was relieved, but the ankles and wrists were severely affected. Light tonics and aperients followed, and the patient left town for the country better, yet with a constitution dreadfully shattered by the long-continued suffering. The change of air did not rally him, and he returned to town, and sought the advice of another physician, under whose care he continued for a month, apparently improving, but not very rapidly. During this time the remedies employed were various; tonics, bitters, aperients, &c. No severe pain was experienced, but occasional pains wandering over the body and limbs were relieved by small doses of Dover's powder.

The patient thought himself able to return to his employment, and essayed to superintend the business of a slaughterhouse: he was tempted to make some violent exertions, and the result was that he was attacked by peritoneal inflammation, that quickly destroyed life.

The post-mortem examination of this case furnished a striking illustration of the extent to which disease may proceed in the production of suffering, without leaving any trace in the structure of the body. The marks of recent inflammation, effusion of serum and floating lymph, were all evident enough to account for death, and of recent origin; but the liver, gallbladder, stomach, and indeed every other organ, were entirely healthy, except a small spot of the stomach, the appearance of which was considered very equivocal. I cannot avoid the conclusion that, had the acetate of lead been given in this case, before the plan adopted in December, it would have removed the primary element of the disease, the nervous pain.

Case IV. Pain in the region of the spleen is common to young unmarried women, whether arising from sympathy with the uterine system, or being a simple nervous affection *per se*, it is by ordinary means very little influenced.

Mary Heygatt, æt. twenty-five, has suffered very greatly for a long time with this pain, aggravated at uncertain intervals, and independent of any mode of living, exercise, or rest. None of the vital or animal functions are at all impaired, except when severe pain produces effects perfectly analogous to those mentioned in Case I. I had twice treated her by bleeding, but the result was so unsatisfactory that I resolved on adopting other means should it again return. The opportunity was soon afforded, and the acetate of lead perfectly removed the pain in two days.

It would be easy for me to add a multiplicity of cases where lead has been beneficially employed. I have selected the foregoing, because they appear distinctly to answer the purpose of standing as exemplars of a great number and variety constantly met with.

In conclusion, I would observe that, should it be supposed, from the absence of particulars, the cases are not sufficiently authenticated, and that this would involve some difficulty in private practice, I should be very happy to satisfy any one on the subject, and also to receive any communications either tending to establish or invalidate my conclusions.

